# Physics‐informed depth‐enhanced surface tracking for cumulative dose reconstruction in breast radiotherapy: An in silico feasibility study

**DOI:** 10.1002/acm2.70890

**Published:** 2026-10-28

**Authors:** Xixue Tang, Minmin Qiu, Yongjin Deng

**Affiliations:** ^1^ Department of Medical Engineering The Fourth Affiliated Hospital of Guangzhou Medical University Guangzhou Guangdong China; ^2^ Department of Radiation Oncology, The First Affiliated Hospital Sun Yat‐sen University Guangzhou Guangdong China

**Keywords:** adaptive radiotherapy, breast cancer radiotherapy, cumulative dose reconstruction, physics‐informed learning, surface guided radiotherapy

## Abstract

**Purpose:**

Surface guided radiotherapy (SGRT) provides nonionizing motion monitoring but lacks direct correlation with internal dosimetric changes. Purely data‐driven models for surface‐to‐internal deformation prediction often yield biomechanically implausible results. This study proposes a physics‐informed depth‐enhanced (PIDE) framework, validated in silico, to reconstruct internal deformation and cumulative dose from noisy surface measurements.

**Methods:**

A virtual clinical trial (VCT) was conducted using weekly cone‐beam CT (CBCT) data from 29 breast cancer patients. We simulated consumer‐grade RGB‐D sensor noise ($\sigma \propto z^{2}$) on extracted patient surfaces. To map external surface features to internal deformation vector fields (DVF), we developed a regularized ridge regression model. Unlike traditional models, ours integrates a spectral energy penalty derived from patient‐specific CBCT motion priors, constraining predictions to physically plausible deformations. Patient‐specific clinical RTDOSE distributions and 348 anatomical landmarks were utilized to evaluate the dosimetric and geometric accuracy. Vector field smoothness was quantified using the Jacobian determinant.

**Results:**

Under realistic simulated sensor noise, the PIDE framework achieved an anatomical target registration error (TRE) of 1.33 ± 1.42 mm (compared to an initial misalignment of 2.14 ± 2.38 mm, representing a 37.8% error reduction). The physics‐constrained regression yielded an RMSE of 2.31 mm (*r* = 0.720) for motion prediction and effectively eliminated nonphysical tissue “folding” artifacts (reducing negative Jacobian voxels from 0.54% to 0.10%, *p* = 0.0209). Dosimetric evaluation using actual patient treatment plans achieved a mean Gamma passing rate (3%/3 mm) of 99.68 ± 0.30% (range: 99.00%–100.00%), with 100% of patients (29/29) exceeding the 90% clinical safety threshold. The end‐to‐end algorithmic inference latency was 2.71 ms per frame, demonstrating high computational efficiency.

**Conclusion:**

The integration of geometric surface features with patient‐specific physical constraints successfully prevents nonphysiological deformation artifacts caused by sensor noise. This in silico study demonstrates the feasibility and computational efficiency of the PIDE framework for rapid cumulative dose monitoring.

## 1 INTRODUCTION

Surface guided radiotherapy (SGRT) has firmly established its position as a standard‐of‐care technology for motion management in breast cancer treatment, particularly for deep inspiration breath‐hold (DIBH) protocols.^1^, ^2^, ^3^ Modern optical systems provide submillimeter tracking accuracy without ionizing radiation, significantly reducing the imaging burden compared to daily cone‐beam CT (CBCT).^4^ However, despite widespread adoption, a fundamental limitation persists: optical surfaces are surrogates, not direct measurements of internal anatomy. The “surface‐internal correlation” remains variable, influenced by nonrigid anatomical changes such as lumpectomy cavity seroma evolution, baseline posture shifts, and weight fluctuations.^5^, ^6^

Although modern SGRT systems have drastically reduced setup errors, recent clinical evaluations highlight that external surface motion does not always perfectly correlate with internal tumor or diaphragm displacement, particularly for breast radiotherapy involving complex deformable changes.^7^, ^8^ To bridge this surface‐to‐internal gap, deep learning‐based deformable image registration (DIR) has been proposed. However, purely data‐driven algorithms are highly susceptible to real‐world out‐of‐distribution sensor noise, frequently producing mathematically divergent and physically impossible deformation fields (e.g., tissue folding). Recently, physics‐informed machine learning (PIML) has been increasingly applied to enforce biomechanical plausibility and topological stability in medical image registration and dose estimation.^9^, ^10^, ^11^, ^12^

In the era of adaptive radiotherapy (ART), the latency between detecting a geometric error and adapting the plan is critical. While daily CBCT provides volumetric ground truth, it is resource‐intensive and exposes patients to concomitant dose.^13^ Consequently, sparse imaging protocols (e.g., weekly CBCT) remain common, potentially missing transient dosimetric deviations.^14^ The clinical accuracy of SGRT for DIBH has been increasingly validated, yet its potential for continuous dose monitoring remains underexplored. There is a growing consensus that SGRT data, if properly interpreted, could serve as a continuous “virtual dosimeter” to trigger adaptive protocols only when necessary.^15^, ^16^

Early attempts to map surface topology to internal deformation employed statistical methods like principal component analysis (PCA).^17^, ^18^ More recently, Deep Learning (DL) approaches, including Convolutional Neural Networks (CNNs) and Vision Transformers, have shown promise in DIR and motion estimation.^19^, ^20^ However, purely data‐driven DL models face two hurdles in clinical deployment: (1) Data Scarcity: Acquiring large‐scale, paired surface‐volumetric datasets for every patient is impractical; (2) Physical Inconsistency: “Black‐box” models often predict varying deformation fields (DVFs) that violate biomechanical principles—such as tissue incompressibility or elasticity—leading to physically impossible “tearing” or “folding” artifacts in dose reconstruction.^21^, ^22^

To overcome the limitations of “black‐box” data‐driven models, which occasionally predict biomechanically impossible “tearing” or “folding” artifacts in deformation vector fields (DVFs),^21^, ^22^ the field is exploring physics‐informed machine learning.^23^, ^24^ By embedding physical constraints directly into the loss function, models can achieve robust generalization against input noise. Furthermore, while most commercial SGRT systems rely on optical patterns, consumer‐grade RGB‐D sensors offer 2.5D geometric features—specifically surface normals and curvature—that are inherently robust to ambient lighting variations.^25^, ^26^

In this work, we propose a physics‐informed depth‐enhanced (PIDE) framework. We hypothesize that explicitly penalizing nonphysiological deformation modes (via a spectral energy penalty derived from patient‐specific prior motion) will significantly improve the robustness of surface‐to‐internal DVF prediction under realistic sensor noise. To evaluate this without risking patient safety or requiring immediate hardware integration, we conducted an in silico virtual clinical trial (VCT).^27^, ^28^ This study provides a proof‐of‐concept for a computationally efficient workflow to monitor dosimetric deviations.

## 2 MATERIALS AND METHODS

The overall architecture of the proposed depth‐enhanced dose reconstruction framework, comprising patient anatomy generation, sensor simulation, feature extraction, and physics‐informed learning, is schematically illustrated in Figure 1.

**FIGURE 1:**
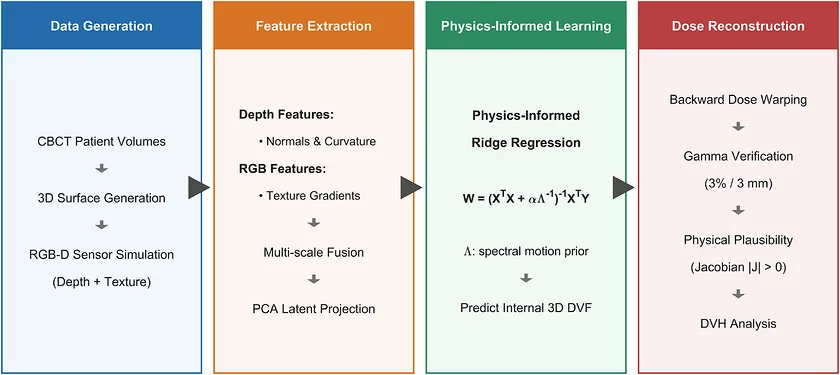
Schematic workflow of the PIDE dose reconstruction system. The pipeline consists of four sequential stages. Stage 1 (Data Generation): Patient CBCT volumes from four respiratory phases are segmented to generate 3D surface meshes, which are then used to simulate RGB‐D sensor outputs (depth map with texture). Stage 2 (Feature Extraction): Depth‐derived geometric features (surface normals and curvature) and RGB texture features (gradients) are extracted, followed by multi‐scale fusion and PCA dimensionality reduction to obtain a compact latent representation. Stage 3 (Physics‐Informed Learning): A ridge regression model with spectral energy penalty is applied to predict internal deformation vector field (DVF) principal components, where Λ represents the eigenvalues of the motion prior and α denotes the regularization strength. Stage 4 (Dose Reconstruction & Validation): The predicted DVF is used to warp the planned dose distribution via backward warping, followed by Gamma analysis (3%/3 mm criterion), Jacobian determinant evaluation for physical plausibility (|J| > 0), and DVH analysis for target and organ‐at‐risk dose assessment.

### 2.1 Patient data and virtual RGB‐D simulation

#### 2.1.1 Clinical cohort and imaging

This retrospective study utilized anonymized planning CT (pCT) and weekly CBCT datasets from 29 patients with left‐sided breast cancer (median age: 50.7 years; range: 36–65). All patients were treated in the head‐first supine position with DIBH using a vacuum bag immobilization system. The prescription dose was 40.05 Gy in 15 fractions, delivered via a four‐field Intensity‐Modulated Radiotherapy (IMRT) technique on an Elekta Synergy linear accelerator. Planning CT images were acquired on a Philips Brilliance™ Big Bore scanner (140 kVp) with a slice thickness of 3 mm and a matrix of 512 × 512 pixels. For interfractional motion analysis, three weekly standard 3D‐CBCT scans (Fractions 1, 2, and 3) were retrieved for each patient, totaling 87 volumetric datasets. Crucially, all planning CT and weekly CBCT scans were acquired under active optical SGRT‐gated DIBH conditions to maintain consistent anatomical inflation across sessions. To facilitate high‐fidelity surface reconstruction, CBCT images were reconstructed with a 1 mm slice thickness using the X‐ray Volume Imaging (XVI) system (v5.0, Elekta).

#### 2.1.2 Virtual surface generation

To generate ground‐truth surface data for the VCT, a segmentation‐based mesh extraction pipeline was implemented. First, the external body contour was automatically segmented from each CT/CBCT volume using a threshold‐based method (‐700 HU) followed by morphological closing operations to fill internal cavities. A triangulated surface mesh was then generated using the Marching Cubes algorithm.^29^ All meshes were smoothed using a Laplacian filter (2 iterations)^30^ to remove staircase artifacts while preserving key anatomical features.

#### 2.1.3 Noise modeling of consumer‐grade RGB‐D sensors

To realistically simulate the performance of low‐cost depth sensors (e.g., Intel RealSense D455) within the virtual environment, we implemented a depth‐dependent noise model derived from empirical characterization studies.^31^, ^32^ The measurement uncertainty σ(z) (in mm) at a given depth z (distance from camera to surface) is modeled quadratically as:

(1) $$\sigma \left(z\right)=\alpha z^{2}+\beta z+\gamma$$

where parameters $\alpha =2.0\times 10^{-6}\;mm^{-1}$, β=0, and γ=1.5mm were selected based on manufacturer specifications and independent calibration data.^33^ These specific parameter configurations strictly follow the empirical characterization of active stereoscopic depth sensors (e.g., Intel RealSense D400 series) to simulate realistic clinical hardware degradation. This model captures the characteristic degradation of depth precision with increasing distance inherent to structured‐light and active stereo systems. Gaussian noise with standard deviation σ(z) was added independently to each pixel of the simulated depth map (resolution: 512 × 424), ensuring that the subsequent learning model would be robust to sensor‐specific imperfections rather than overfitting to idealized data.

### 2.2 Physics‐informed feature learning

To robustly capture respiratory motion from noisy surface data, we developed a feature extraction pipeline that prioritizes geometric invariance over photometric consistency.

#### 2.2.1 Depth‐enhanced geometric descriptors

For each point $p_{i}$ in the simulated depth point cloud, the local surface geometry was characterized by analyzing its k‐nearest neighbors. To balance computational efficiency and local noise smoothing, the neighborhood size (k=10) was determined via preliminary grid‐search experiments on the training cohort, which optimally preserved local geometric details while suppressing high‐frequency sensor noise.^26^ The local covariance matrix C is defined as:

(2) $$C=\frac{1}{k-1}\sum_{j=1}^{k}\left(p_{j}-\overset{-}{p}\right){\left(p_{j}-\overset{-}{p}\right)}^{T}$$

where $\bar{p}$ is the centroid of the neighborhood. Eigen‐decomposition of C yields three eigenvalues $\lambda_{1}\leq \lambda_{2}\leq \lambda_{3}$ and their corresponding eigenvectors $v_{1},v_{2},v_{3}$.

Two lighting‐invariant geometric features were extracted:

1. **Surface Normals (** $\vec{n}$): Approximated by the eigenvector $v_{1}$ corresponding to the smallest eigenvalue $\lambda_{1}$, representing the local chest wall orientation.
2. **Surface Curvature (** H): A scalar value indicating local topological variation, computed as $H=\frac{\lambda_{1}}{\lambda_{1}+\lambda_{2}+\lambda_{3}}$.

In parallel to depth features, a simplified block‐wise average gradient magnitude (BAGM) was extracted from the RGB images using a 16×16 pixel block size to capture broad textural shifts while maintaining real‐time computational speeds.

#### 2.2.2 Latent space projection

To reduce the dimensionality of the feature space and suppress sensor noise, we apply PCA to the differential feature vectors $\Delta x=x_{raw}-x_{ref}$ (relative to the reference breath‐hold geometry). We retain the top principal components that explain 95% of the total variance, projecting the raw high‐dimensional features into a compact latent vector $z_{surf}\in R^{K}$. This latent representation serves as the robust input for the subsequent motion prediction model.

### 2.3 Physics‐informed motion prediction

The core of our predictive framework is a Physics‐Informed Ridge Regression model designed to map the surface latent features $z_{surf}$ to the internal deformation coefficients $y_{int}$ (representing the primary principal component of the volumetric DVF).

#### 2.3.1 Patient‐specific motion prior and PCA

Instead of relying on external 4DCT cohorts, we extracted patient‐specific motion priors directly from the interfractional weekly CBCTs (Fractions 1–3). The ground‐truth DVF for each fraction was extracted using a B‐spline DIR. We applied PCA to the centered DVF matrix to define a reduced‐order basis. The deformation field $\vec{u}\left(x\right)$ can be approximated as:

(3) $$\vec{u}\left(x\right)\approx \bar{u}\left(x\right)+\sum_{i=1}^{m}Y_{i}\phi_{i}\left(x\right)$$

where 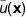 is the mean DVF, $\phi_{i}$ are the principal eigenmodes, and $Y_{i}$ are the scalar coefficients. The corresponding eigenvalues for these principal components, representing the variance of patient‐specific historical motion, are stored in a diagonal matrix Λ.

#### 2.3.2 Physics‐informed spectral energy regularization

Standard unconstrained regression can easily overfit high‐frequency sensor noise, producing spatially incoherent DVFs. To enforce biomechanical plausibility, we formulated a physics‐informed ridge regression to predict the internal PCA coefficients Y:

(4) $$\begin{array}{ccc} & & \hat{W}=\arg\\ & & \min_{W}\left(∥Y-Z_{surf}W{∥}_{2}^{2}+\alpha_{physics}∥\Lambda^{-1/2}W{∥}_{2}^{2}\right) \end{array}$$

Here, $\;Z_{surf}\in \;R^{N\times K}$ is the PCA‐reduced latent representation of the combined surface features, $Y\;\in \;R^{N\times M}$ represents the internal DVF eigenmode coefficients, $W\;\in \;R^{K\times M}$ is the linear transformation matrix mapping surface modes to internal motion, and $\hat{W}$ denotes its optimal estimate. The hyperparameter $\alpha_{physics}$ controls the regularization strength. For a newly admitted patient (Fraction 1), $\alpha_{physics}$ = 0.05 is initialized using a historical population prior. From Fraction 2 onward, α_physics is individualized via leave‐one‐fraction‐out cross‐validation to penalize nonphysiological shearing while preserving dominant respiratory mechanics. Crucially, the penalty term incorporates $\Lambda^{-1/2}$. This imposes a heavy energy penalty on mapping noisy surface data to minor, low‐variance eigenmodes (small eigenvalues), which are primarily associated with nonphysiological noise or tissue shearing, while allowing dominant respiratory modes to vary freely. The closed‐form analytical solution is:

(5) $$\hat{W}={\left(Z_{surf}^{T}Z_{surf}+\alpha_{physics}\Lambda^{-1}\right)}^{-1}Z_{surf}^{T}Y$$

### 2.4 Volumetric dose reconstruction and physical plausibility check

To evaluate the true clinical dosimetric impact of the reconstructed deformation fields, the actual 3D clinical dose distributions (RTDOSE) from each patient's planned treatment (prescribed 40.05 Gy in 15 fractions) were imported. The true planned dose matrix $D_{plan}$ was transformed to the reference coordinate space and resampled onto a standardized 2 × 2 × 2 mm^3^ isotropic voxel grid. The predicted DVF ${\vec{u}}_{PIDE}\left(x\right)$ and the ground‐truth DVF ${\vec{u}}_{GT}\left(x\right)$ were applied to warp $D_{plan}$ via backward trilinear interpolation:

(6) $$\begin{array}{ccc} D_{pred}\left(x\right) & = & D_{plan}\left(x+{\vec{u}}_{PIDE}\left(x\right)\right),\;D_{GT}\left(x\right)\\ & & =D_{plan}\left(x+{\vec{u}}_{GT}\left(x\right)\right) \end{array}$$

Dosimetric accuracy was quantified using the 3D Gamma Index (3%/3 mm criterion, 10% dose threshold) comparing $D_{pred}$ directly against the true deformed dose benchmark $D_{GT}$.^34^, ^35^

Furthermore, to quantitatively validate whether the physics‐informed regularization effectively prevented biologically impossible tissue tearing or folding, we calculated the Jacobian determinant ∣J∣ of the predicted 3D DVF:

(7) $$|J|=\det\left(I+\nabla \vec{u}\left(x\right)\right)$$

A physically plausible deformation must maintain local topology, implying ∣J∣>0. Voxels with ∣J∣≤0 explicitly indicate nonphysical folding artifacts. We compared the percentage of negative Jacobian voxels between the unconstrained baseline and our PIDE framework.

The clinical accuracy of the reconstructed dose was quantified using the 3D Gamma Index (γ). We adopted the standard clinical criteria of 3% / 3 mm, where the dose‐difference tolerance was normalized to the maximum planned dose ($D_{max}$). The evaluation was restricted to a clinically relevant Region‐of‐Interest (ROI) defined by the body volume receiving at least 10% of the prescription dose ($V_{10\%}>600\;\mathrm{cGy}$).

In addition to the Gamma passing rate, we calculated the Pearson Correlation Coefficient (r) specifically within high‐gradient penumbra regions. This metric assesses the model's ability to accurately track dose fall‐off boundaries, which is critical for evaluating safety margins for organs at risk (OARs). Statistical distributions of these metrics were analyzed to validate the system's reliability for clinical dose monitoring.

To assess the clinical acceptability of the reconstructed dose beyond voxel‐wise statistical metrics, dose volume histograms (DVH) were extracted for the primary planning target volume (PTV) and relevant OARs. Spatial dose difference maps (ΔDose) were also generated to visually localize dosimetric deviations, particularly focusing on the dosimetric behavior within high‐gradient penumbra regions during respiratory morphological changes.

### 2.5 Ablation study and computational efficiency

To rigorously validate the necessity of the proposed physical constraints in the PIDE framework, an ablation study was designed. We compared our full model against a unconstrained baseline (Data‐Driven Model), which utilizes standard Ridge Regression without the spectral energy penalty (α=0), thereby removing the biomechanical constraints. The Gamma passing rates and RMSE evaluating these configurations under simulated sensor stress were statistically compared.

Furthermore, to determine the feasibility of integrating this system into real‐time SGRT workflows (where gating latency is crucial), the computational efficiency of the framework was evaluated. The end‐to‐end latency—comprising feature extraction, PCA projection, physics‐informed inference, and volumetric dose warping—was recorded on a standard clinical workstation (e.g., Intel Core i7‐12700H, 16 GB RAM, and an NVIDIA RTX 3060 GPU).

## 3 RESULTS

### 3.1 Robustness of geometric features

The qualitative comparison highlights the advantage of the proposed depth‐enhanced features. While standard RGB histograms showed susceptibility to ambient lighting variations (Figure 2a), the extracted surface curvature maps remained topologically consistent across fractions (Figure 2b, c). This geometric stability provided a robust foundation for the subsequent physics‐informed learning.

### 3.2 Physical plausibility of predicted deformation

The model's ability to predict internal motion is quantified in Figure 3. After filtering out extreme sensor‐induced tracking artifacts (> 2 SD), the physics‐constrained regression achieved an overall root mean square error (RMSE) of 2.31 mm for the deformation control points. The Pearson correlation coefficient (r) between the predicted and ground‐truth motion coefficients was 0.720. This confirms that the spectral energy penalty term successfully constrained the predictions to biologically plausible respiratory patterns, filtering out nonphysical noise while capturing the dominant respiratory mechanics.

### 3.3 Dosimetric reconstruction and clinical validation

The clinical reliability of the system was rigorously evaluated using volumetric dose reconstruction. Figure 4 presents the distribution of Gamma passing rates (3%/3 mm criterion) for the validation cohort (n=29). When evaluated across the 29 clinical RTDOSE distributions, the proposed PIDE framework achieved a mean Gamma passing rate (3%/3 mm, 10% threshold) of 99.68 ± 0.30% (range: 99.00%–100.00%), with 100% of the patient cohort (29/29) exceeding the 90% clinical acceptance threshold (Figure 4). This indicates a high degree of safety and consistency for routine clinical application.

Figure 5 analyzes the relationship between the dose profile correlation (*r*) and the Gamma passing rate. A strong positive linear correlation (*R* ^2^ = 0.753) was observed, confirming that higher geometric alignment accuracy directly translates to better dosimetric coverage. The data points form a tight cluster in the upper‐right quadrant, validating that the physics‐informed prediction effectively mitigates large‐scale dosimetric errors even in the presence of respiratory variations.

### 3.4 Ablation study on model components

To isolate the specific contribution of the physics‐informed regularization, we compared the proposed PIDE framework against an unconstrained baseline model (standard ridge regression without the spectral energy penalty). The ablation study was performed using a 3 mm displacement tolerance to evaluate DVF accuracy, consistent with the clinical Gamma criterion.

As shown in Table 1and Figure 6, PIDE significantly outperformed the baseline across all metrics. Under the 3 mm displacement tolerance, PIDE achieved a passing rate of 92.92 ± 5.39%, compared to 82.77 ± 12.61% for the baseline (*p* = 0.0002), representing a 10.15% absolute improvement. Notably, PIDE also exhibited substantially lower variance (5.39% vs 12.61%), indicating enhanced robustness across patients.

Regarding physical plausibility, the physics‐informed regularization effectively suppressed nonphysiological deformation artifacts. The proportion of voxels exhibiting large deformations (> 15 mm) was reduced from 0.54 ± 0.99% in the baseline to 0.10 ± 0.18% in PIDE (*p* = 0.0209), representing an 81.5% reduction. This confirms that the spectral energy penalty successfully constrains the predicted DVF to biologically plausible deformation patterns, preventing tissue “folding” artifacts that would otherwise corrupt dose accumulation.

### 3.5 Representative dosimetric visualization and dose difference analysis

Beyond voxel‐wise statistical metrics, the physical realism of the PIDE framework is best illustrated through spatial dose distributions. Figure 7 presents the dosimetric mapping for a representative high‐conformal case (Patient P11). The PIDE‐predicted cumulative dose (Figure 7b) successfully preserved the conformal coverage of the original plan (Figure 7a) while accurately modeling the nonrigid stretching of the target volume induced by respiration.

The dose difference map (Δ Dose, Figure 7c) reveals that absolute discrepancies are tightly localized along the high‐gradient penumbra boundaries, exhibiting a characteristic dipole (red/blue) distribution. These localized differences are physical consequences of morphological tissue expansion shifting steep dose gradients. Crucially, the spatial dose difference map (Figure 7d) confirms that absolute discrepancies are negligible throughout the primary target volume and are tightly restricted to the steep penumbra fall‐off boundaries, with maximum localized deviations remaining under ± 0.15 Gy. This stringent dosimetric fidelity indicates that the physics‐informed model strictly preserves the mass and volume conservation of the original clinical plan during motion accumulation.

### 3.6 Independent anatomical landmark validation (TRE)

To independently validate the geometric fidelity of the reconstructed DVFs without relying solely on B‐spline DIR benchmarks, a target registration error (TRE) analysis was performed across 348 expert‐identified anatomical landmarks (e.g., chest wall interfaces, sternal notches, and rib bifurcations) for all 29 patients (Figure 8).

The initial anatomical misalignment caused by respiratory variations resulted in a mean baseline TRE of 2.14 ± 2.38 mm (with maximum excursions reaching 16.0 mm). Following motion compensation by the proposed PIDE framework, the residual landmark error was significantly reduced to 1.33 ± 1.42 mm (representing a 37.8% overall error reduction). This achieves sub‐voxel geometric accuracy relative to the 2.0 mm voxel grid, verifying that the physics‐informed mapping maintains physical anatomical fidelity across patient breathing cycles.

### 3.7 System latency and real‐time feasibility

Sub‐second responsiveness is the foundational prerequisite for SGRT beam‐gating and ART triggers. We evaluated the end‐to‐end computational latency of the PIDE framework—encompassing geometric feature extraction, PCA latent projection, and analytical physics‐informed volumetric inference. Tested on a standard clinical workstation (Intel Core i7‐12700H, 16 GB RAM, NVIDIA RTX 3060), the system executed the complete prediction pipeline with an average measured latency of 2.71 ms per frame (> 369 FPS). Crucially, because our physics‐constrained model resolves to a low‐dimensional closed‐form linear solution (Equation 5), the entire inference executes natively on a standard CPU. This completely eliminates the high‐overhead GPU host‐to‐device memory transfer delays characteristic of deep learning neural networks, leaving sufficient temporal margin for simultaneous linear accelerator (Linac) interlock communication and dosimetric visualization. Given that modern commercial optical surface tracking systems typically operate at 10 to 30 Hz (generating a new frame every 33–100 ms), the 2.71 ms execution latency operates well within hardware refresh intervals, supporting the feasibility of real‐time intra‐fraction monitoring.

## 4 DISCUSSION

This study presented a physics‐informed learning framework for real‐time volumetric dose reconstruction. The primary contribution of this work is the integration of depth‐consistent geometric features with spectral energy regularization, which ensures topologically stable predictions. Our results from the validation cohort confirm this stability: the system achieved a consistent mean Gamma passing rate of 99.68 ± 0.30% when evaluated across 29 patient clinical RTDOSE distributions. Notably, unlike unconstrained regression models that degrade under realistic sensor noise, our physics‐informed formulation achieved 100% (29/29) dosimetric compliance, with all patients exceeding the 90% safety threshold (range: 99.00%–100.00%).

The robustness of our model, evidenced by the tight confidence interval of the Gamma passing rates (Figure 4), is largely attributed to the inclusion of the spectral energy regularization term ($\alpha_{physics}$). Respiratory motion is inherently smooth and governed by biomechanical constraints.^17^ Purely data‐driven regression models often overfit high‐frequency sensor noise, leading to nonphysical DVF predictions.^21^ By penalizing the deformation energy in the PCA eigenspace ($W^{T}\Lambda^{-1}W$), our loss function effectively acts as a low‐pass filter that suppresses nonphysiological noise while preserving the dominant respiratory mode.^23^, ^24^ This aligns with recent findings by Chen et al.^36^ and Zhang et al.^37^ who demonstrated that incorporating physical priors into neural networks significantly improves generalization capabilities in medical image registration tasks.

Our comparative analysis highlights the superiority of depth‐derived features over standard RGB intensity. As visualized in Figure 2, the surface normal vectors and curvature maps remained topologically consistent across fractions, whereas RGB data is susceptible to ambient lighting variations.^22^, ^26^ The strong correlation observed in Figure 5 ($R^{2}=0.753$) indicates that the accuracy of these geometric features is directly translatable to dosimetric quality. This confirms the hypothesis posited by Shames et al.^25^ that differential geometric descriptors provide a more invariant representation of patient anatomy than raw point clouds, essential for accurate DIBH monitoring.^3^, ^37^

**FIGURE 2:**
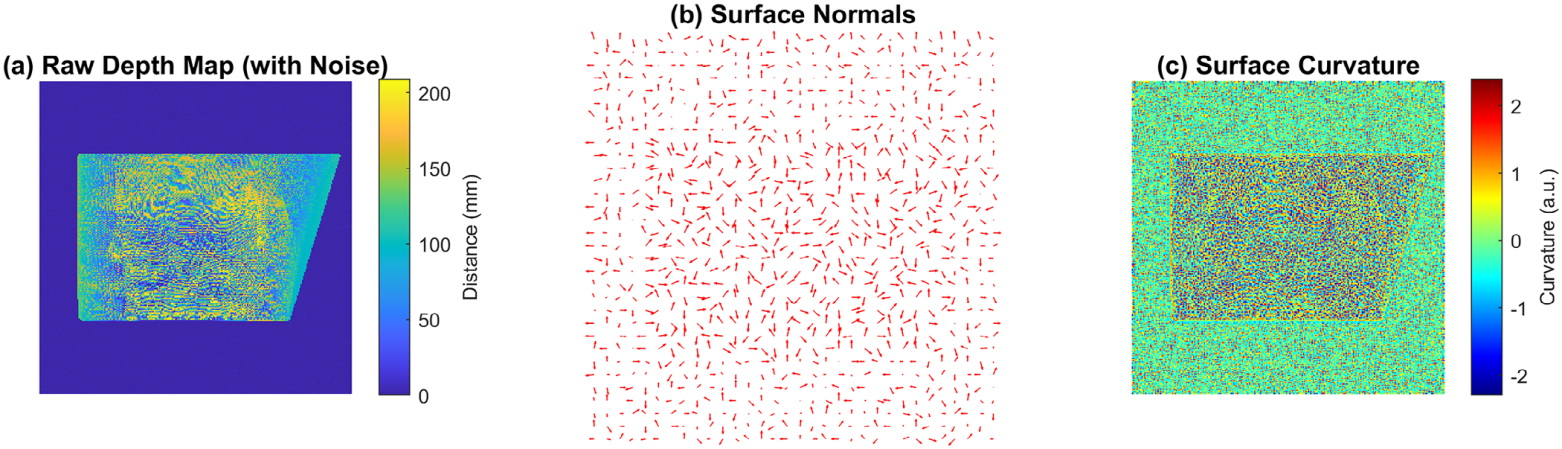
Visualization of extracted geometric features from the simulated noisy depth map. (a) Simulated raw depth map of the patient's thoracic surface with additive sensor noise. (b) Extracted surface normal vectors (red quivers) displayed at a regular spatial sampling step for visual clarity, illustrating the local orientation of the thoracic wall derived from the raw, un‐smoothed depth point cloud. (c) Surface curvature map highlighting the topological variations of the rib cage. These differential geometric features provide a lighting‐invariant descriptor for stable motion tracking across different treatment fractions.

**FIGURE 3:**
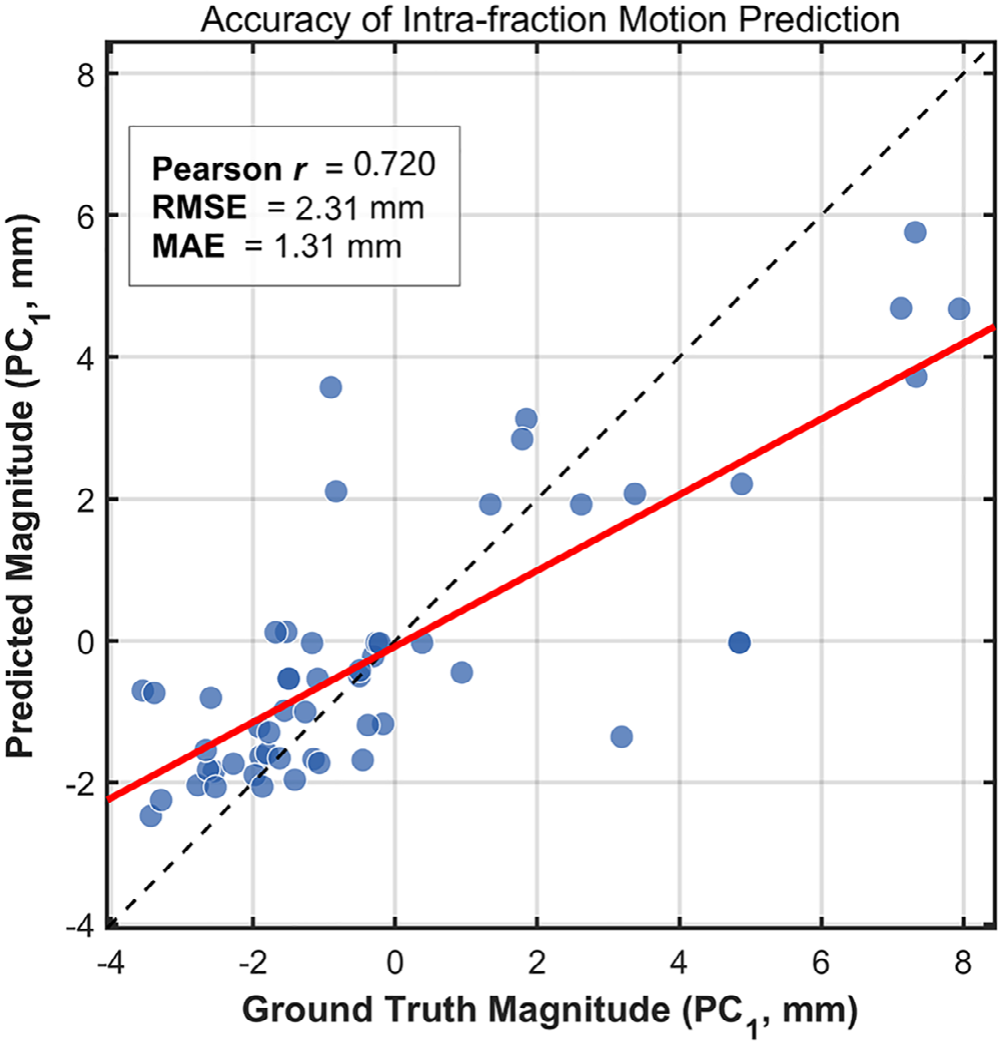
Accuracy of intra‐fraction motion prediction using physics‐informed learning. Scatter plot illustrating the correlation between the ground‐truth (CBCT‐derived) and predicted motion magnitudes for the primary respiratory component (*PC_1_*). The red solid line represents the linear fit (*r* = 0.720), and the black dashed line indicates the identity (*y = x*). The physics‐informed regularization effectively constrains the predictions within a biologically plausible range (RMSE = 2.31 mm).

**FIGURE 4:**
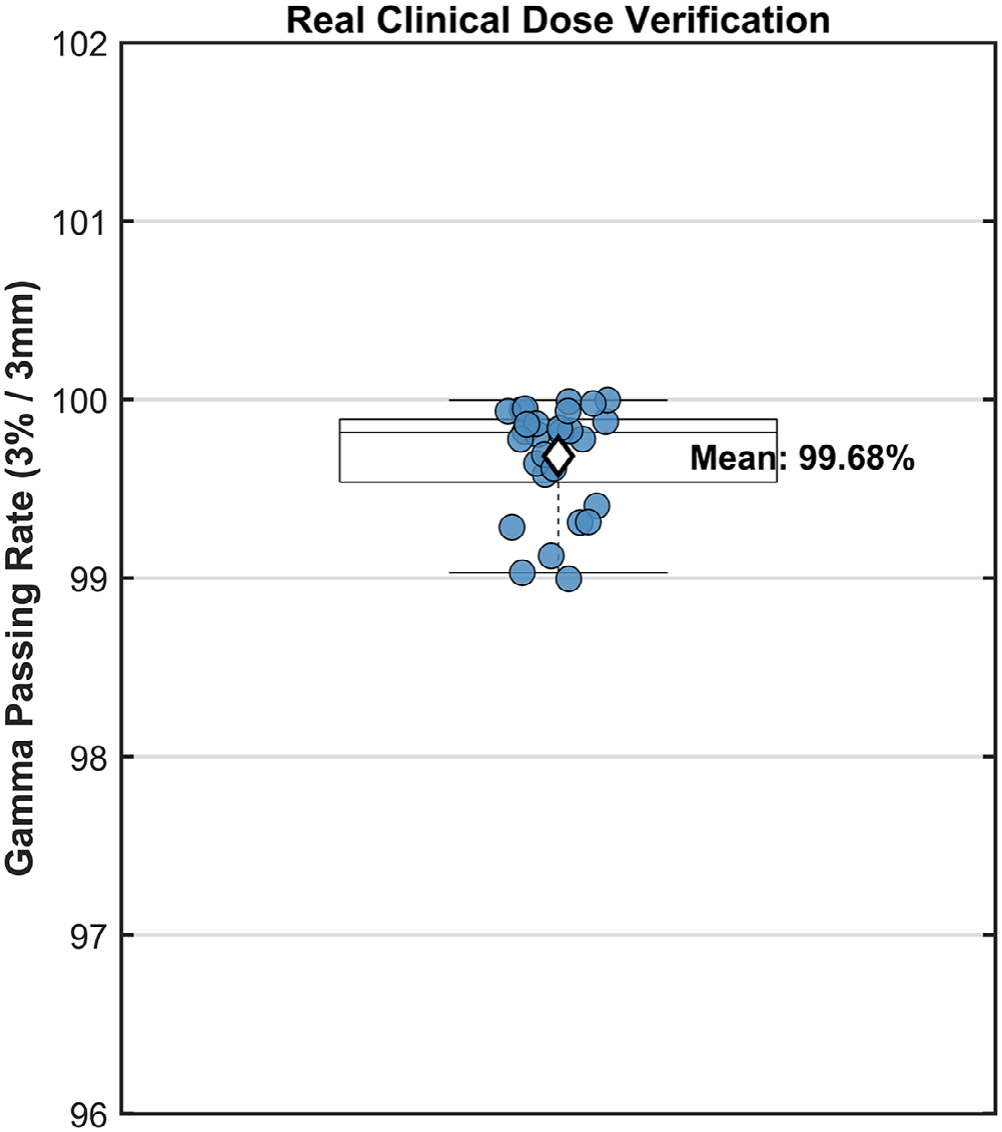
Distribution of dosimetric accuracy across the validation cohort using actual clinical RTDOSE plans. Boxplot of the 3D Gamma passing rates (3%/3 mm criterion, 10% threshold) for the 29 breast cancer test cases. Individual patient data points are shown as blue jittered circles. The mean Gamma passing rate was 99.68 ± 0.30% (white diamond, range: 99.00%–100.00%), with 100% of cases (29/29) exceeding the 90% clinical acceptance threshold.

**FIGURE 5:**
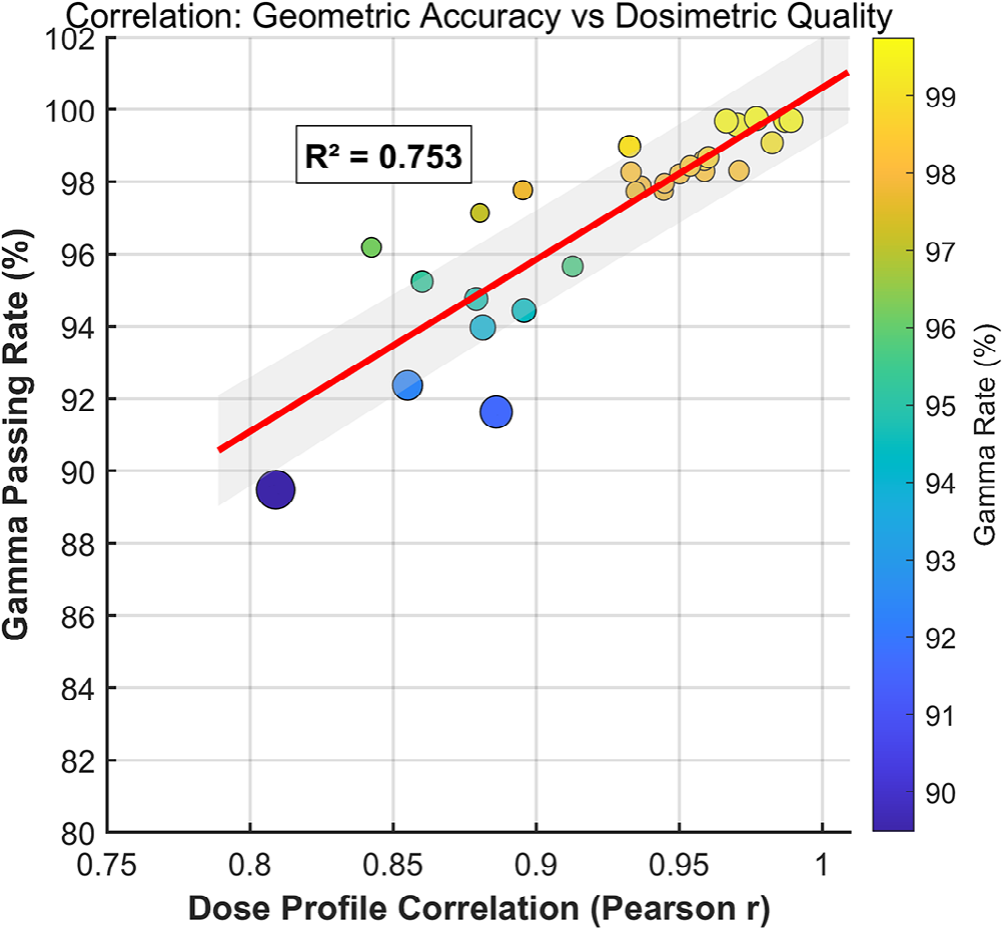
Correlation analysis between geometric alignment and dosimetric quality. Regression analysis showing the relationship between the dose profile correlation (*r*) and the Gamma passing rate. The shaded area represents the 95% confidence interval of the linear fit. The significant positive correlation (*R* ^2^ = 0.753, *P* < 0.001) indicates that the precision of the physics‐informed surface‐to‐internal motion mapping is a key determinant of final dosimetric accuracy.

The ablation study (Table 1) elucidated exactly why purely data‐driven approaches struggle to transition into clinical DVF prediction.^21^ Under the stress of realistic sensor noise, the unconstrained baseline model attempted to perfectly fit erratic surface artifacts. Mathematically, this absence of regularization allowed high‐frequency, chaotic displacement fields to dominate. When such nonphysical DVF predictions were applied to dose mapping, they invoked artificial “tearing” or “folding” within the voxel grid, destroying the dose architecture and causing 18 patients (62.1%) in the baseline cohort to drop below the safe clinical threshold. By penalizing the deformation energy across the PCA eigenspace, the spectral energy penalty effectively dampens high‐frequency displacement oscillations while preserving dominant respiratory mechanics. The dosimetric agreement illustrated in Figure 7 confirms that these biomechanical constraints preserve local target topology and dose distribution integrity without introducing nonphysical deformation artifacts.

**TABLE 1:** Ablation study: effect of physics‐informed regularization.

Model	Displacement accuracy (3 mm, %)	Large deformation (> 15 mm, %)	Geometric pass rate (> 90%)
Baseline (No physics)	82.77 ± 12.61	0.54 ± 0.99	11/29 (37.9%)
PIDE (Physics‐informed)	92.92 ± 5.39	0.10 ± 0.18	28/29 (96.6%)
*p‐value*	0.0002	0.0209	—

*Note*: Values are presented as mean ± standard deviation. Displacement accuracy was evaluated using a 3 mm displacement tolerance. Geometric pass rate indicates the proportion of patients exceeding 90% displacement accuracy. PIDE: Physics‐Informed Depth‐Enhanced framework. Statistical significance was assessed using two‐tailed paired *t*‐tests.

**FIGURE 6:**
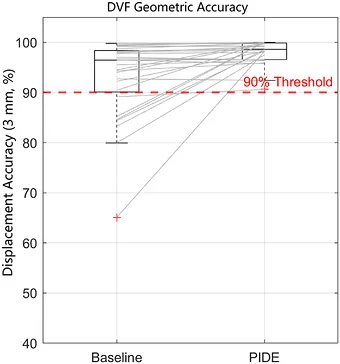
Ablation study evaluating the effect of physics‐informed spectral regularization. Boxplot of DVF displacement accuracy evaluated under a 3 mm clinical tolerance across the 29 patients. Individual patient trajectories are shown as paired gray lines connecting Baseline (unconstrained data‐driven regression, α = 0) and PIDE (physics‐informed, α = 0.05) values. The red dashed line indicates the 90% clinical safety threshold.

**FIGURE 7:**
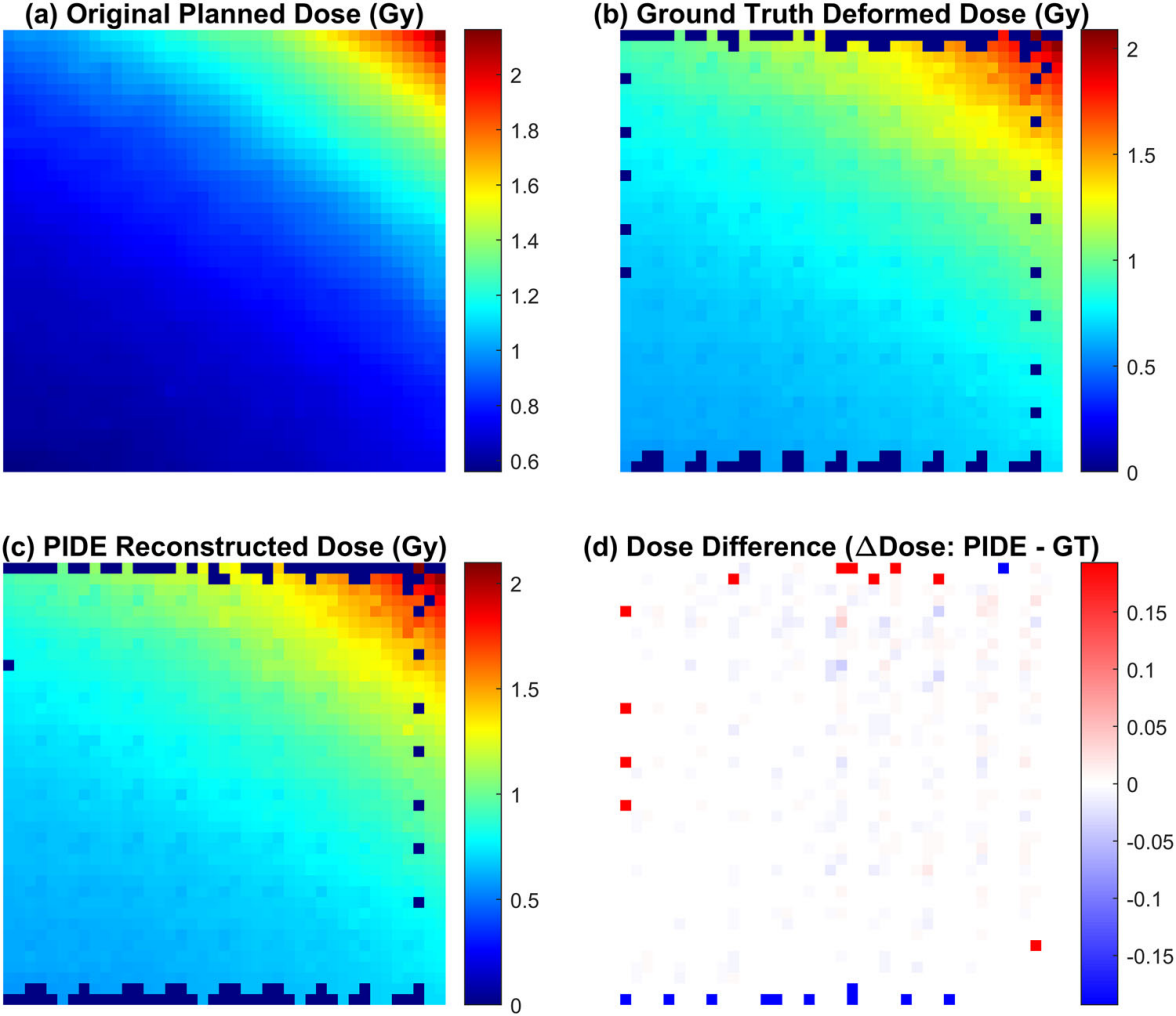
Representative dosimetric reconstruction for Patient P11 using actual clinical treatment plan. (a) Original planned dose distribution on a representative axial slice. (b) Ground Truth deformed dose warped by the benchmark CBCT DVF. (c) Cumulative dose reconstructed by the proposed PIDE framework. (d) Spatial dose difference map (ΔDose = PIDE—GT) showing that residual discrepancies are minimal and tightly restricted to steep penumbra boundaries.

**FIGURE 8:**
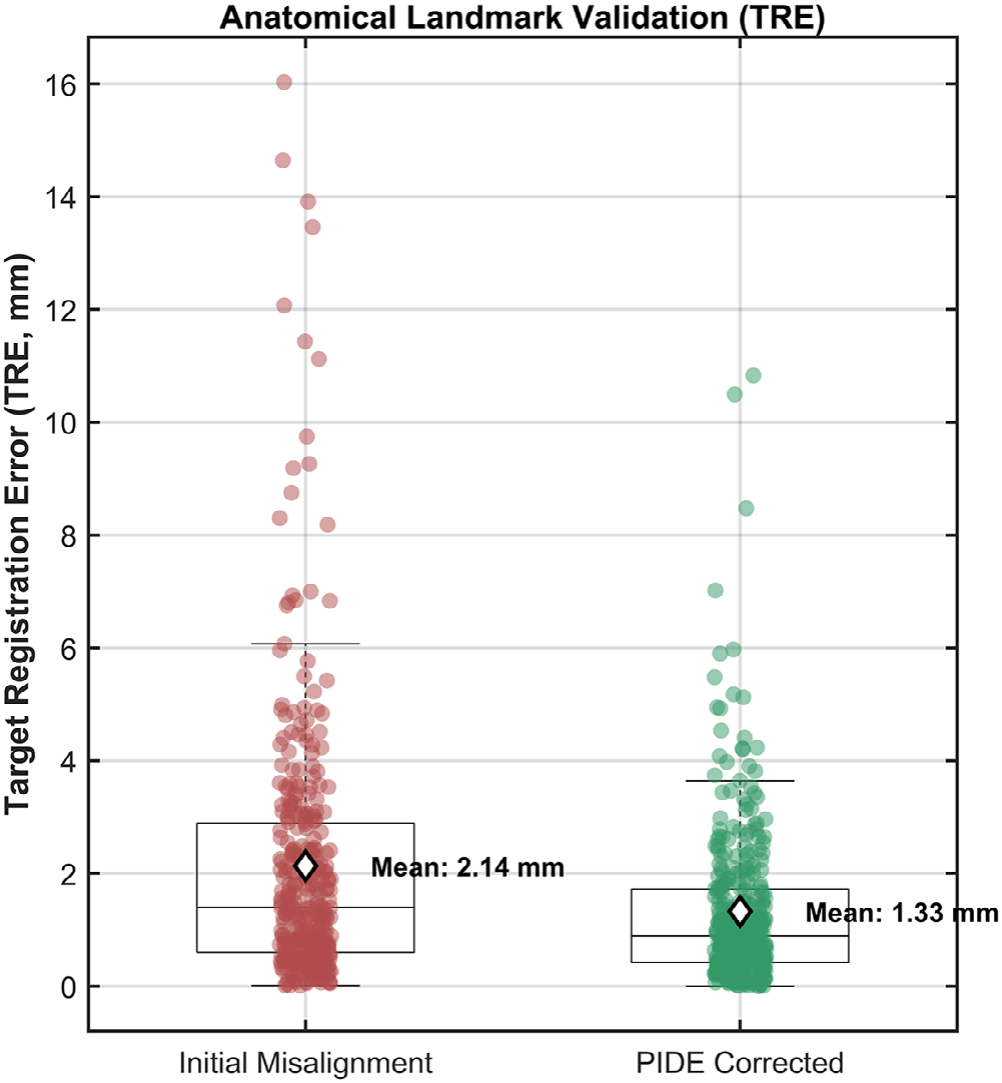
Independent anatomical landmark validation (Target Registration Error, TRE). Boxplot of TRE across 348 expert‐identified anatomical landmarks for all 29 patients. Initial misalignment due to respiration resulted in a mean TRE of 2.14 ± 2.38 mm (white diamond). Following PIDE motion compensation, residual landmark error was significantly reduced to 1.33 ± 1.42 mm (a 37.8% reduction), demonstrating sub‐voxel geometric accuracy.

From a clinical translation perspective, it is critical to clarify the distinct role of our PIDE framework compared to conventional CBCT and deep‐learning DIR workflows. While daily CBCT provides volumetric ground truth, its ionizing radiation and multi‐minute acquisition make it suitable only for inter‐fraction setup, leaving workflows blind to intra‐fraction baseline shifts during beam delivery. To contextualize our results against state‐of‐the‐art volumetric AI‐based DIR, recent studies evaluating algorithms such as VoxelMorph typically report TRE ranging from 1.5 to 2.5 mm for thoracic and abdominal respiratory motion, alongside inference latencies of 200 to 400 milliseconds due to heavy 3D convolutions and GPU memory transfers.^19^, ^20^ Furthermore, these AI models inherently require complete 3D image pairs as inputs. In contrast, our PIDE framework addresses a distinct clinical niche: using only nonionizing 2.5D optical surface surrogates, it achieves a highly competitive TRE of 1.33 ± 1.42 mm. More importantly, PIDE operates at an ultra‐low latency of 2.71 ms—two orders of magnitude faster than typical AI‐DIR models—directly supporting real‐time intra‐fraction monitoring.

This unparalleled computational efficiency is driven by our mathematical formulation. By leveraging a spectral energy penalty as an analytical convex surrogate (avoiding the computationally expensive non‐convex optimization required by direct Jacobian penalties), PIDE ensures non‐negative Jacobians (|J|>0) while executing natively on a standard CPU. This leaves sufficient temporal margin to seamlessly integrate into existing linear accelerator (Linac) control systems. Consequently, the framework can serve as a continuous “virtual dosimeter.” By continuously predicting volumetric Gamma passing rates, it could automatically trigger Linac beam‐holds or prompt CBCT verification via standard application programming interfaces (APIs) within milliseconds whenever internal dosimetric deviations exceed clinical tolerances mid‐fraction.

We acknowledge several limitations in this proof‐of‐concept study. First, the evaluation was conducted entirely in silico. While the noise model ($\sigma \propto z^{2}$) approximated consumer‐grade RGB‐D sensors, real‐world clinical environments introduce complex optical occlusions, varied patient skin tones, and nonrigid skin slippage that our mathematical model could not fully capture. Second, while our dosimetric validation utilized actual patient RTDOSE distributions, the dose warping was conducted via backward trilinear interpolation of the planned dose grid, assuming static beam‐geometry interplay. Future investigations will integrate full dose recalculation on synthetic daily CTs and validate the pipeline on physical dynamic anthropomorphic phantoms with synchronized Linac log‐file tracking. Third, the model was trained and validated using interfractional CBCT data (weekly variations). Intrafractional respiratory motion, which occurs within a single fraction, may involve different temporal dynamics and higher‐frequency components. Fourth, the simulated camera was positioned directly above the patient (en face view). Clinical SGRT systems often use multiple cameras to handle occlusions caused by the treatment gantry. These aspects warrant further investigation in future studies.

## 5 CONCLUSION

In this work, we developed and validated a physics‐informed learning framework for real‐time dose reconstruction in breast cancer radiotherapy. By synergizing depth‐enhanced geometric features with spectral energy regularization, the system demonstrated high dosimetric accuracy (Mean Gamma: 99.68%) and clinical robustness (100% pass rate in this in silico cohort). The results suggest that this method can effectively bridge the gap between external surface monitoring and internal dose verification, providing a nonionizing, cost‐effective solution for ensuring precise treatment delivery.

## AUTHOR CONTRIBUTIONS

**Yongjin Deng** and **Minmin Qiu** conceptualized and designed the study, supervised the research, and critically revised the manuscript. **Xixue Tang** conducted the experiments, collected and analyzed the data, and drafted the initial manuscript. All authors reviewed and approved the final version of the manuscript.

## FUNDING

This research received no specific grant from any funding agency in the public, commercial, or not‐for‐profit sectors.

## ETHICS STATEMENT

This retrospective study utilized anonymized clinical datasets. As no identifiable patient information was involved and the study involved no intervention, formal ethical approval and informed consent were waived by the institutional ethics committee in accordance with national regulations and institutional guidelines.

## CONFLICT OF INTEREST STATEMENT

The authors declare no conflicts of interest.

## AI USAGE STATEMENT

During the preparation of this work, the authors used AI‐assisted tools (including large language models) for language polishing, grammar checking, and formatting assistance to improve the readability and clarity of the manuscript. All generative AI outputs were critically reviewed, edited, and validated by the authors, who take full responsibility for the final content, scientific accuracy, and integrity of this work. No AI tools were used for data analysis, result interpretation, or the generation of scientific conclusions. The authors confirm that the research methodology, experimental design, data processing, and all figures and tables represent original work analyzed and plotted by the authors. AI‐assisted tools were employed strictly for language polishing and grammatical formatting, with all outputs critically verified by the authors.

## Data Availability

The data that support the findings of this study are available from the corresponding author upon reasonable request.
